# Comparison of Metacognitive Therapy Versus Cognitive Behavioral Therapy for Generalized Anxiety Disorder: A Meta-Analysis of Randomized Control Trials

**DOI:** 10.7759/cureus.39252

**Published:** 2023-05-20

**Authors:** Anurag Rawat, Niraj Sangroula, Areeba Khan, Sana Faisal, Ali Chand, Rao Ahmed Yousaf, Nazar Muhammad, Humayoun Yousaf

**Affiliations:** 1 Interventional Cardiology, Himalayan Institute of Medical Sciences, Dehradun, IND; 2 Psychiatry, College of Medical Sciences, Bharatpur, NPL; 3 Critical Care Medicine, United Medical and Dental College, Karachi, PAK; 4 Internal Medicine, California Institute of Behavioral Neurosciences & Psychology, Fairfield, USA; 5 Internal Medicine, Jinnah Sindh Medical University, Karachi, PAK; 6 Medicine, Shaikh Khalifa Bin Zayed Medical and Dental College, Lahore, PAK; 7 Psychiatry, Faisalabad Medical University, Faisalabad, PAK; 8 Psychiatry, Cornerstone Family Healthcare, New York, USA

**Keywords:** efficacy, meta-analysis, generalized anxiety disorder, cognitive behavioral therapy, metacognitive therapy

## Abstract

The aim of this meta-analysis is to compare the efficacy of meta-cognitive therapy (MCT) and cognitive behavioral therapy (CBT) in patients with generalized anxiety disorder (GAD). This study is reported according to the guidelines of Preferred Reporting Items for Systematic Reviews and Meta-Analyses (PRISMA). A systematic electronic literature search was conducted on April 20, 2023, to find studies reporting on the efficacy of MCT for GAD. The search keywords included “Generalized anxiety disorders,” “meta-cognitive therapy,” “cognitive behavior therapy,” and “randomized control trials.: The following databases were searched to find relevant articles: PubMed, PsychInfo, CINAHL, and SCOPUS. Outcomes assessed in the present meta-analysis included the change in the Penn State Worry Questionnaire (PSWQ) from baseline to completion of treatment and after two years of follow-up. The PSWQ measures the trait of worry in adults. Worry is regarded as a dominant feature of GAD. Secondary outcomes assessed in this meta-analysis included symptom severity using the Beck anxiety inventory (BAI). Change in BAI was scored from baseline to completion of treatment and after two years of follow-up. A total of three studies were included in this meta-analysis. The results show that patients treated with MCT had greater reductions in PSWQ and BAI scores post-treatment and after two years of treatment, as well as higher rates of recovery compared to those treated with CBT. These findings suggest that MCT is a promising approach for treating GAD and may have advantages over traditional CBT approaches.

## Introduction and background

Generalized anxiety disorder (GAD) is a prevalent condition that has long-lasting and negative impacts on one’s quality of life [1]. The main features of GAD are persistent, unmanageable worries that relate to various events or actions lasting for at least six months. GAD is often accompanied by physical symptoms such as restlessness, tiredness, poor concentration, irritability, sleep difficulties, and muscle tension [2]. GAD is associated with increased rates of impairment, comorbidity, low quality of life, and long-term disability [3-5].

The preferred form of psychotherapy for GAD is cognitive behavioral therapy (CBT) [6]. CBT has been shown to bring about significant improvement in GAD, with around 50% of patients experiencing clinical improvement two years after treatment [7,8]. Metacognitive therapy (MCT) is a newer approach to treating GAD that has proven to be particularly successful [9]. Research has demonstrated that in randomized trials for anxiety and depression, MCT resulted in recovery rates ranging from 72% to 80% [9]. MCT is an alternative therapy option to CBT. MCT emphasizes changing thought processes instead of thought content [10]. It is a good comparator for CBT as it appears to be effective and does not involve exposure, cognitive restructuring, breathing techniques, or applied relaxation, which are at the core of CBT, thus decreasing overlap [11].

MCT was developed as a way of improving the effectiveness of treatment for various mental health conditions, including GAD [12]. MCT is based on the metacognitive model of GAD, which emphasizes the significance of metacognitive beliefs regarding worry in maintaining the disorder. Positive metacognitions involve beliefs that worrying is necessary, while negative metacognitions include beliefs about the uncontrollability and harmfulness of worrying [13]. MCT aims to modify unhelpful metacognitive beliefs rather than the content of worrying itself. Numerous clinical trials have demonstrated that MCT is a successful treatment for various disorders, including GAD [10].

The previous meta-analysis conducted by Normann et al. in 2018 assessed the efficacy of MCT involving patients with depression, anxiety, and other psychological illnesses. The study found that MCT is highly efficient in decreasing symptoms of a range of primary targeted psychological complaints, along with symptoms of depression, anxiety, and maladaptive metacognitions. However, the present meta-analysis focuses on the effectiveness of MCT in patients with GAD and included recently conducted clinical trials as well [10]. Our meta-analysis aims to compare the efficacy of MCT with CBT in patients with GAD.

## Review

Methodology

This study was reported according to the guidelines of Preferred Reporting Items for Systematic Reviews and Meta-Analyses (PRISMA). A systematic electronic literature search was conducted on April 20, 2023, to find studies reporting on the efficacy of MCT for GAD. The search keywords included “Generalized anxiety disorders,” “meta-cognitive therapy,” “cognitive behavioral therapy,” and “randomized control trials.” We used Medical subject headings (MeSH) terms along with boolean algebra operators to further enhance the search. The following databases were searched to find relevant articles: PubMed, PsychInfo, CINAHL, and SCOPUS. The reference lists of included articles were also manually screened.

Studies were included in this meta-analysis if (a) they were randomized controlled trials (RCTs) on adult patients with GAD; (b) they compared the efficacy of MCT with CBT; and (c) they were published in the English language. Studies were excluded if they were retrospective and prospective cohorts, case-control studies, reviews, case reports, and case series. We excluded studies that did not report desired outcomes, cross-over studies, and those that lacked a comparison group.

Data Abstraction and Risk of Bias Assessment

The present meta-analysis was performed according to the PRISMA 2020 guidelines. The titles and abstracts of all studies from the four databases that matched the keywords were separately screened according to the eligibility criteria and duplicates were removed. Two reviewers performed the literature search (AR, NS), screening, and eligibility assessments (SF, AC). Abstracts and titles were screened separately according to the above-mentioned inclusion criteria. Disagreements between the two reviewers were resolved by discussion. Two reviewers extracted data from included studies such as author name, year of publication, sample size, intervention, outcomes assessed, follow-up duration, and participants’ characteristics. The risk of bias of all included studies was calculated using the Cochrane Risk of Bias assessment tool. The risk of bias was assessed by two authors independently.

Outcomes assessed in this meta-analysis included the change in the Penn State Worry Questionnaire (PSWQ) from baseline to completion of treatment and after two years of follow-up. The PSWQ measures the trait of worry in adults. Worry is regarded as a dominant feature of GAD. Secondary outcomes assessed in this meta-analysis included symptom severity using the Beck anxiety inventory (BAI). Change in BAI was scored from baseline to completion of treatment and after two years of follow-up. The number of patients recovered (as defined by individual studies) after completion of the treatment was also assessed as a secondary outcome in this meta-analysis.

Data Synthesis and Analysis

A meta-analysis was performed using RevMan 5.4.1 (The Cochrane Collaboration, London, United Kingdom). For continuous outcomes, we calculated the mean difference (MD) with a 95% confidence interval (CI). For categorical outcomes, the risk ratio (RR) was computed with a 95% CI. A p-value <0.05 was considered significant. To assess the variability in the study outcomes, we employed the I^2^ statistic, which indicates the percentage of variation between studies that results from differences other than chance. It has been proposed that I^2^ values of 25%, 50%, and 75% can be classified as indicating low, moderate, and high degrees of diversity, respectively.

Results

**Figure 1:**
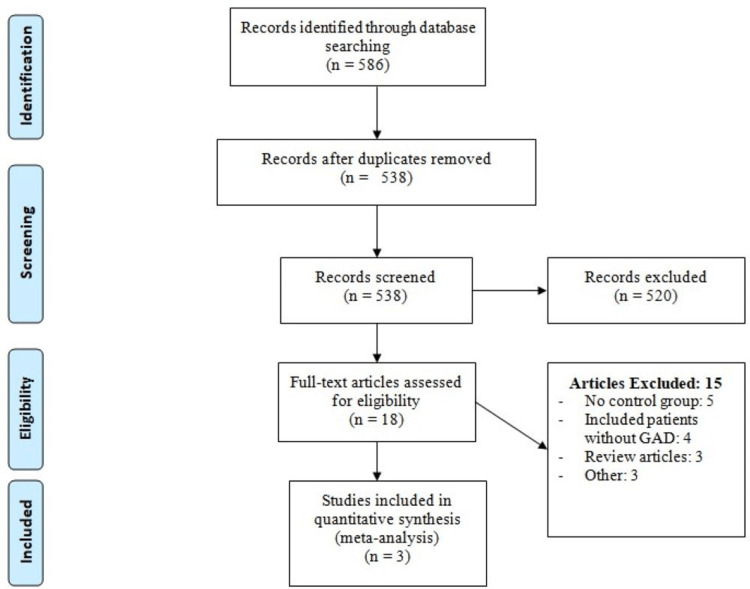
PRISMA flowchart of selection of studies. GAD = generalized anxiety disorder; PRISMA = Preferred Reporting Items for Systematic Reviews and Meta-Analyses

**Figure 2:**
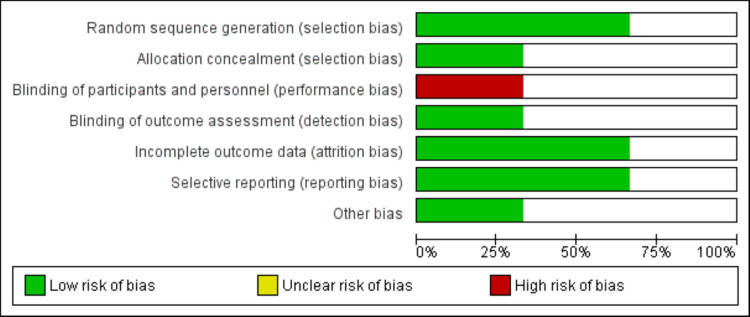
Risk of bias graph.

We identified 586 records through online database searching. After removing duplicates, 538 articles were screened using titles and abstracts. The full text of eligible records was obtained and a detailed assessment was done based on the pre-defined inclusion and exclusion criteria. Finally, three RCTs met the eligibility criteria and were included in this meta-analysis. Figure 1 shows the process of study selection. Table 1 shows the characteristics of included studies. Figure 2 illustrates the risk of bias graph.

**Table 1:** Characteristics of included studies. CBT = cognitive behavior therapy; MCT = metacognitive therapy

Author name	Year	Groups	Sample size	Therapy	Follow-up
Heiden et al. [14]	2012	MCT	54	14 weekly sessions of 45 minutes duration	6 months
		CBT	52		
Nordahl et al. [11]	2018	MCT	32	12 weekly sessions of 60 minutes duration	2 years
		CBT	28		
Solem et al. [15]	2021	MCT	22	12 weekly sessions of 60 minutes duration	2 years
		CBT	17		

Meta-Analysis of Outcomes

**Figure 3:**
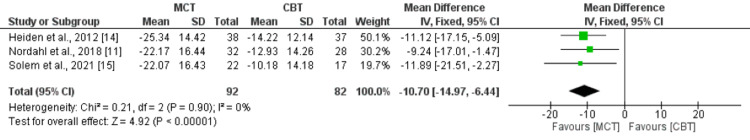
Comparison of change in the PSWQ score from baseline to completion of treatment. MCT = metacognitive therapy; CBT = cognitive behavioral therapy; PSWQ = Penn State Worry Questionnaire Sources: references [11,14,15].

**Figure 4:**
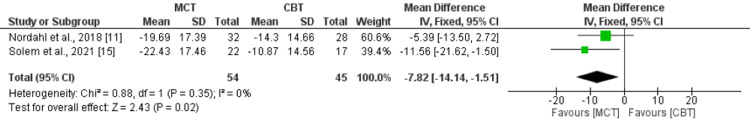
Comparison of change in the PSWQ score from baseline to two years post-treatment. MCT = metacognitive therapy; CBT = cognitive behavioral therapy; PSWQ = Penn State Worry Questionnaire Sources: references [11,15].

The difference in the PSWQ score from baseline to post-treatment was assessed by three studies. Pooled analysis showed that the reduction in the PSWQ score post-treatment was significantly higher in patients randomized in the MCT group compared to patients randomized in the CBT group (MD = -10.70, 95% CI = -14.97, -6.44, p < 0.0001), as shown in Figure 3. No significant heterogeneity was reported among the study results (I^2^ = 0%). Two studies assessed the change in the PSWQ score from baseline to the two-year follow-up. Pooled analysis showed that the reduction in the PSWQ score post-treatment was significantly higher in patients randomized in the MCT group compared to patients randomized in the CBT group (MD = -7.82, 95% CI = -14.14, -1.51, p < 0.02), as shown in figure 4. No significant heterogeneity was reported among the study results (I^2^ = 0%).

**Figure 5:**
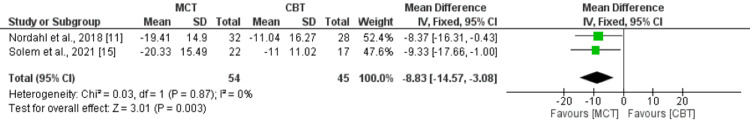
Comparison of change in the BAI score from baseline to completion of treatment. MCT = metacognitive therapy; CBT = cognitive behavioral therapy; BAI = Beck anxiety inventory Sources: references [11,15].

**Figure 6:**
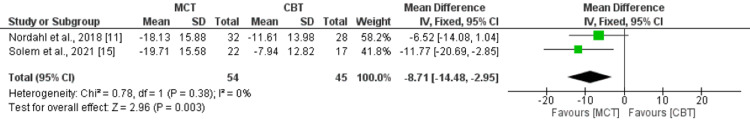
Comparison of change in the BAI score from baseline to two years post-treatment. MCT = metacognitive therapy; CBT = cognitive behavioral therapy; BAI = Beck anxiety inventory Sources: references [11,15].

Two studies assessed change in BAI from baseline to post-treatment. Pooled analysis showed that the reduction in the BAI score was significantly greater in patients receiving MCT (MD = -8.83, 95% CI = -14.57, -3.08), as shown in Figure 5. No significant heterogeneity was reported among the study results (I^2^ = 0%). We also assessed the change in the BAI score from baseline to the two-year follow-up. Pooled analysis showed that the reduction in the BAI score was significantly higher in patients receiving MCT from baseline to the two-year follow-up (MD = -8.71, 95% CI = -14.48, -2.95), as shown in Figure 6. No significant heterogeneity was reported among the study results (I^2^ = 0%).

Recovery Rates

Three studies compared recovery rates between the MCT and CBT groups. After completion of treatment, pooled rate of recovery was significantly higher in patients receiving MCT (67.36%) compared to patients receiving CBT (42.86%) (RR = 1.59, 95% CI = 1.19, 2.11). No heterogeneity was reported among the study results (I^2^ = 0%).

**Figure 7:**
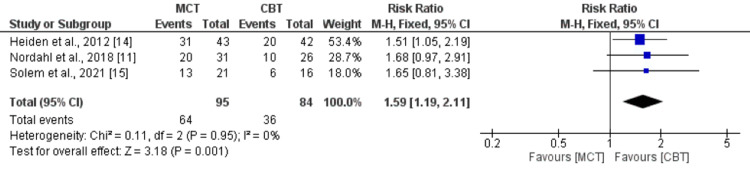
Comparison of the effect on MCT and CBT on recovery rates. MCT = metacognitive therapy; CBT = cognitive behavioral therapy

Discussion

In this meta-analysis, we aimed to compare the efficacy of MCT and CBT in patients with GAD. We included three clinical trials, and our pooled analysis showed that MCT is more effective in reducing PSWQ and BAI scores. Additionally, the proportion of recovered patients was significantly greater in the MCT-treated group compared to the other study group. This is the first meta-analysis comparing MCT and CBT in patients with GAD.

The benefit of using MCT instead of CBT was observed at the end of treatment and at a medium-term follow-up. This advantage was still present at a two-year follow-up. The difference in results at the long-term follow-up may be because the two therapies address different psychological mechanisms. CBT aims to teach relaxation skills and challenge the content of worry, whereas MCT focuses on challenging beliefs about worry without addressing the content of worry. MCT helps patients regulate worry processes in a way that reduces the significance of thoughts. It is possible that the better outcome of MCT is due to greater improvement in dysfunctional metacognitions that form the foundation of long-term mental regulation [16].

MCT is having a significant impact on the treatment of various psychiatric disorders worldwide, particularly in the areas of major depression and anxiety disorders, which have been the focus of most research so far [17,18]. The transdiagnostic approach of MCT makes it easy to adapt and requires less time to gain mastery, which makes it appealing to psychotherapists. MCT is well-suited to the current situation of psychiatric disorders, where comorbidity is common, and comorbid disorders share similar underlying psychological substrates and psychosocial vulnerabilities [19,20]. MCT can simultaneously address symptoms of multiple psychiatric disorders instead of treating them one at a time, as done in sequential approaches such as CBT. Additionally, MCT is shorter in duration than CBT, which can require 12-20 sessions [21]. As a result, it can lead to rapid improvement, reduce disease-related distress and dysfunction, and decrease the overall cost of treatment while freeing up the therapist’s time to attend to more patients. MCT has been found to be not only feasible and acceptable (as indicated by high treatment adherence) but also effective in treating various psychiatric disorders [22].

This study has certain limitations. First, only three studies were included in the pooled analysis. However, all studies support the use of MCT in patients with GAD. Second, we were not able to perform subgroup analysis based on the severity of the disease. To enhance conclusions about the efficacy of MCT, we advise future MCT trials to include randomized control designs with evidence-based comparison conditions, particularly when looking at anxiety or depression.

## Conclusions

This meta-analysis indicates that MCT may be more effective than CBT in the treatment of GAD. The results show that patients treated with MCT had greater reductions in PSWQ and BAI scores and higher rates of recovery compared to those treated with CBT. These findings suggest that MCT is a promising approach for treating GAD and may have advantages over traditional CBT approaches. However, it is important to note that the sample size of studies included in this analysis was small, and further studies with larger sample sizes are needed to confirm these findings. Nonetheless, the transdiagnostic approach and shorter duration of MCT make it a feasible and cost-effective option for the treatment of GAD and other psychiatric disorders. Future studies should focus on the efficacy of MCT in treating more severe cases of GAD and compare it to other evidence-based treatments. Overall, this study contributes to the growing body of research on MCT and its potential as a valuable alternative to traditional therapies for the treatment of GAD.
